# Single Cell Scale Neuronal and Glial Gene Expression and Putative Cell Phenotypes and Networks in the Nucleus Tractus Solitarius in an Alcohol Withdrawal Time Series

**DOI:** 10.3389/fnsys.2021.739790

**Published:** 2021-11-19

**Authors:** Sean J. O’Sullivan, Damani McIntosh-Clarke, James Park, Rajanikanth Vadigepalli, James S. Schwaber

**Affiliations:** ^1^Department of Pathology, Anatomy, and Cell Biology, Daniel Baugh Institute for Functional Genomics and Computational Biology, Thomas Jefferson University, Philadelphia, PA, United States; ^2^Brain Stimulation Lab, Department of Psychiatry and Behavioral Sciences, Stanford University, Stanford, CA, United States; ^3^Department of Emergency Medicine, Icahn School of Medicine at Mount Sinai, New York, NY, United States; ^4^Department of Chemical Engineering, University of Delaware, Newark, DE, United States; ^5^Institute for Systems Biology, Seattle, WA, United States

**Keywords:** alcohol withdrawal, neuroinflammation, RT-PCR, subphenotypes, single-cell heterogeneity, microglia

## Abstract

Alcohol withdrawal syndrome (AWS) is characterized by neuronal hyperexcitability, autonomic dysregulation, and severe negative emotion. The nucleus tractus solitarius (NTS) likely plays a prominent role in the neurological processes underlying these symptoms as it is the main viscerosensory nucleus in the brain. The NTS receives visceral interoceptive inputs, influences autonomic outputs, and has strong connections to the limbic system and hypothalamic-pituitary-adrenal axis to maintain homeostasis. Our prior analysis of single neuronal gene expression data from the NTS shows that neurons exist in heterogeneous transcriptional states that form distinct functional subphenotypes. Our working model conjectures that the allostasis secondary to alcohol dependence causes peripheral and central biological network decompensation in acute abstinence resulting in neurovisceral feedback to the NTS that substantially contributes to the observed AWS. We collected single noradrenergic and glucagon-like peptide-1 (GLP-1) neurons and microglia from rat NTS and measured a subset of their transcriptome as pooled samples in an alcohol withdrawal time series. Inflammatory subphenotypes predominate at certain time points, and GLP-1 subphenotypes demonstrated hyperexcitability post-withdrawal. We hypothesize such inflammatory and anxiogenic signaling contributes to alcohol dependence via negative reinforcement. Targets to mitigate such dysregulation and treat dependence can be identified from this dataset.

## Introduction

**FIGURE 1:**
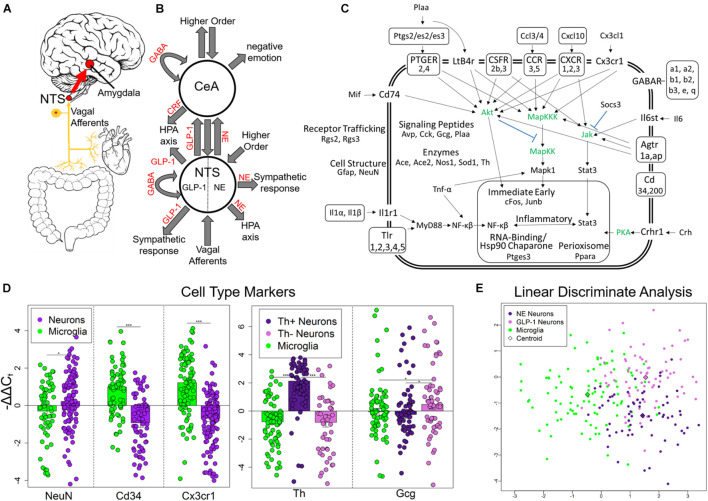
Experimental design and single-cell selection. **(A)** Nucleus tractus solitarius (NTS) is primary viscerosensory nucleus of brain that relays inputs to limbic system via the central nucleus of the amygdala (CeA). Originally published in O’Sullivan and Schwaber (2021). **(B)** Diagram displaying NTS and CeA functions in emotion, stress, and autonomic regulation. NTS GLP-1 and NE neurons are highlighted. Many anatomical and functional connections are omitted for clarity (NE, norepinephrine; GLP-1, glucagon-like peptide 1; GABA, γ-aminobutyric acid; HPA axis, hypothalamic-pituitary-adrenal axis; CRF, corticotropin releasing hormone). Originally published in O’Sullivan and Schwaber (2021). **(C)** Cell diagram displaying genes assayed and their function. Genes in green were not assayed. Official gene symbol is used. **(D)** Gene expression of cell type markers. Error bars show standard error. Neurons compared to microglia *p*-values = 0.0273, 3.94E-10, 7.73E-12, respectively. Th+ neurons showed elevated Th expression compared to Th− neurons (*p* = 4.56E-11) and microglia (*p* = 2.95678E-15). Th− neurons showed elevated expression of Gcg compared to Th+ neurons (*p* = 0.0106) and microglia (*p* = 0.0435) indicating they are GLP-1+ neurons. *p < 0.05, ***p < 4E-10. **(E)** Linear discriminate analysis of all samples displays the difference across all genes measured between the three cell types collected in a two-dimension space. Centroid distance between NE neurons and GLP-1 neurons = 3.30, NE neurons and microglia = 1.57, GLP-1 Neurons and microglia: 2.92.

Alcohol withdrawal syndrome (AWS) is characterized by adverse physical and emotional symptoms. Physical symptoms are driven by autonomic dysregulation, γ-aminobutyric acid (GABA) hypoactivity, and increased glutamatergic signaling leading to dysphoria, nausea, diaphoresis, tachycardia, hypertension, seizures, and delirium tremens (Kosten and O’Connor, 2003). Fear and anxiety are the principle emotional symptoms. The negative reinforcement hypothesis of substance dependence postulates that these negative physical and emotional symptoms experienced in withdrawal motivate alcohol dependence (Koob and Le Moal, 2001, 2008b; Baker et al., 2004; Retson et al., 2015). We conjecture that peripheral network decompensation is a central facet of this model, and that neurovisceral feedback via the vagus nerve conveying peripheral information to the central nervous system contributes substantially to the severity of symptoms experienced (O’Sullivan and Schwaber, 2021) (Figures 1A,B). Investigation into the underlying mechanisms producing these symptoms may provide insight into targets that mitigate acute and protracted AWS severity and prevent relapse following abstinence. Such treatments may provide clinical utility for other substances of abuse with severe withdrawal syndromes such as opioids.

Neuroinflammatory processes have emerged as an important contributor to the severity of AWS symptoms, especially in the amygdala (Koob, 2009; McBride et al., 2010; Freeman et al., 2012a, 2013; Whitman et al., 2013; Breese and Knapp, 2016; O’Sullivan et al., 2019; Roberto et al., 2020; O’Sullivan and Schwaber, 2021). The amygdala is strongly implicated in threat detection and negative emotion, and inflammation here may drive some of the fear and anxiety experienced in AWS (Phelps and LeDoux, 2005; Yang et al., 2016; O’Sullivan and Schwaber, 2021). The likely mechanism underlying this phenomenon is that inflammation causes neuronal hyperexcitability (Schäfers and Sorkin, 2008).

The nucleus tractus solitarius (NTS) is another brain region that contributes to the symptoms of AWS and is implicated in alcohol dependence (King et al., 1991; Covarrubias et al., 2005; Bär et al., 2006; McDonald et al., 2008; Herman, 2012; Aimino et al., 2018). The NTS receives visceral inputs from the interoceptive vagal circuit, strongly influences autonomic outputs, and has strong bidirectional connections to the amygdala (Figure 1A). Connections to the paraventricular nucleus, ventrolateral medulla, and amygdala place the NTS in the center of the visceral-emotional neuraxis (Figure 1B) (Holt and Trapp, 2016; Maniscalco and Rinaman, 2018; O’Sullivan and Schwaber, 2021). Many of these connections use the neuropeptide glucagon-like peptide-1 (GLP-1) as a transmitter. Indeed, interoceptive vagal afferents synapse onto GLP-1 positive (+) NTS neurons that go on to form anxiogenic synapses (Han et al., 1986; Larsen et al., 1997; Rinaman, 1999; Gu et al., 2013; Zheng et al., 2015). Recently, GLP-1R activity in the NTS has been linked to alcohol-mediated behavior (Vallöf et al., 2019). Additionally, the NTS houses noradrenergic (NE) neurons that also respond to vagal and higher-order inputs and principally function to maintain cardiovascular homeostasis (Figure 1B). These NE+ neurons also project to the amygdala where they contribute to emotional memory (Kalia et al., 1985; Williams et al., 1998; Ferry et al., 1999; Maniscalco and Rinaman, 2018). We conjecture a model in which GLP-1 and NE neurotransmission form parallel complementary pathways conveying the peripheral state via NTS to the limbic forebrain (Figures 1A,B).

Indeed, NE+ and GLP-1+ neurons in the NTS have been shown to contribute to symptoms of AWS and alcohol intake in withdrawal (Koob, 2014; Jerlhag, 2020). Further, inflammatory glial-neuronal signaling in the NTS during AWS may also contribute to the severity of physical and emotional withdrawal symptoms (Freeman et al., 2012a, 2013; O’Sullivan and Schwaber, 2021). Local inflammatory signaling in the NTS contributes to the development of hypertension in rats implicating paracrine cytokine involvement in AWS (Waki et al., 2010; DeCicco et al., 2015). Moreover, the anti-neuroinflammatory molecule ibudilast is in clinical trials to reduce alcohol craving and AWS severity (Ray et al., 2017; Grodin et al., 2021).

Here, we measured how the functional states of single-neuron samples containing neuronal phenotypes enriched with NE neurons or GLP-1 neurons and microglia in the NTS change over the course of alcohol withdrawal. Single-cell approaches allow for the identification of cellular subphenotypes—morphologically indistinguishable cells anatomically localized that use the same primary neurotransmitter yet have distinct transcriptomic profiles. Our previous work has demonstrated the heterogeneity of single-cells, and the functional importance of subphenotypes that may be missed in tissue-level approaches (Park et al., 2014, 2016). Changes in transcription during alcohol withdrawal revealed a pattern suggesting peak dysregulation and inflammation at the 32-hour (h) withdrawal (wd) time point. Additionally, the expression of GABA_*A*_ receptor (R) subunits genes was downregulated in protracted withdrawal, measured as the 176 h wd time point, suggesting hyperexcitability of anxiogenic GLP-1 enriched neuronal samples.

## Results

We used laser capture microdissection (LCM) to gather single cells from rat NTS in control, chronic ethanol (EtOH), 8- h wd, 32 h wd, or 176 h wd treatments (Supplementary Figure 1A) 0.10 cells were pooled to comprise a sample that underwent microfluidic reverse transcription quantitative polymerase chain reaction (RT-qPCR) to measure a subset of the transcriptome in these samples comprising 10 individually selected single-cells (Supplementary Figure 1B) (O’Sullivan et al., 2020). Following strict quality control protocols, a total of 229 10-cell pooled samples (700 NE neurons, 650 GLP-1 neurons, and 940 microglia) and 65 gene transcripts were used for data analysis (Figure 1C and Supplementary Tables 1, 2). We targeted gene transcripts involved in inflammatory glial-neuronal signaling and GABA_*A*_R subunits. Cellular phenotype selection was validated by the expression of the cell type markers *NeuN*, *Cd34*, and *Cx3cr1* (Figure 1D). Neurons were selected based on NEUN and TH immunofluorescence. TH positivity can indicate any catecholamine neuron—dopamine (DA), NE, or epinephrine. However, previous studies have demonstrated that the NTS houses NE neurons specifically (i.e., the A2 cell column) and few other catecholamine neurons (Armstrong et al., 1982; Kalia et al., 1985; Reiner and Vincent, 1986). TH- neurons demonstrated significantly elevated levels of the *Gcg* transcript (Figure 1D). This transcript is a precursor for eight peptides, one of which is GLP-1 which has demonstrated an important role in anxiogenic neurotransmission from the NTS to the amygdala (Holt and Trapp, 2016). Accordingly, these TH- neuronal samples were labeled as a phenotype enriched with GLP-1 neurons. A dimensionality reduction analysis (linear discriminate analysis) further demonstrated the differences between the three cell types gathered. Microglia differed from neurons along the x-axis, samples enriched with NE neurons and GLP-1 neurons differed from each other along the y-axis (Figure 1E).

**FIGURE 2:**
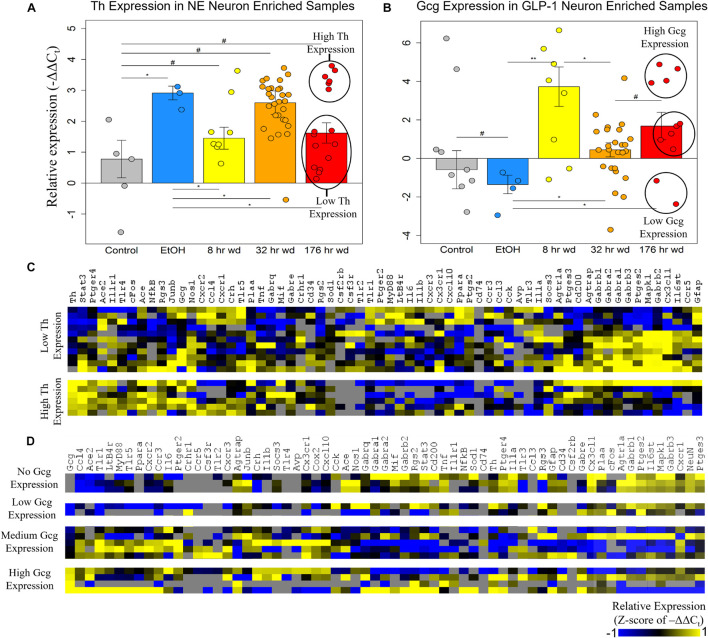
Neurotransmitter Expression in Neurons. **p < 0.003 *p < 0.05, #*p* < 0.1; two-tailed heteroscedastic *t*-test. Bars show standard error. **(A)** Tyrosine hydroxylase expression (Th) in norepinephrine (NE) neuron enriched samples. Bimodal Th expression at the 176 h wd time point is explored in the heat map in panel **(C)**. **(B)** Preproglucagon (Gcg) expression in GLP-1 neuron enriched samples. Trimodal Gcg expression at the 176 h wd time point is explored in the heat map in panel **(D)**. **(C)** Heat map exploring high Th-expressing neurons and low Th-expressing neurons as subclusters of NE neuron enriched samples in the 176 h wd treatment. Single-cell gene expression is shown as z-scores of –ΔΔC_t_ values. Neurotransmitter expression levels does not determine high-template co-expression gene clusters. **(D)** Heat map exploring high, middle, and low Gcg-expressing neurons as subclusters of GLP-1 enriched neuronal samples in the 176 h wd treatment. Single-cell gene expression is shown as z-scores of –ΔΔC_t_ values. Neurotransmitter expression levels does is more predictive of co-expression gene clusters than Th.

The expression of the neurotransmitter precursor genes *Th* and *Gcg* for NE and GLP-1 neuron enriched samples, respectively, is plotted across treatment time points in Figures 2A,B. We find that expression of these transcripts is inversely correlated—at time points in which *Th* expression is relatively high in NE neuron enriched samples, *Gcg* expression is relatively low in GLP-1 neuron enriched samples, and vice versa (Figures 2A,B). We speculate this may indicate a push-pull dynamic mechanism in the genetic regulation of neurotransmission by these neurons. Notably, *Gcg* expression was induced in the three withdrawal time points though moderately at the 32 h wd time point, in which *Th* expression was induced, consistent with the aforementioned push-pull dynamic. Additionally, *Gcg* was low in control and EtOH treatments which may suggest that GLP-1 neurotransmission is pathologically elevated during the withdrawal process. These hypothesis-generating observations require verification. At the 176 h wd time point, bimodal distribution of *Th* expression in NE neuron enriched samples and trimodal *Gcg* expression in GLP-1 neuron enriched samples is observed (Figures 2A,B). High and low *Th*-expressing NE neuron enriched samples and *Gcg*-expressing GLP-1 neuron enriched samples from this time point were separated into heat maps organized by Euclidian distance clustering of gene expression (Figures 2C,D). *Th* expression in NE neurons and *Gcg* expression in GLP-1 neurons is moderately predictive of cellular subphenotypes that loosely organize co-expression gene clusters. However, *Th*-expression and *Gcg*-expression alone, which is to say neurotransmitter expression, is not the best determinate of cellular subphenotypes—a finding we have observed previously in other neuronal nuclei (Park et al., 2016).

**FIGURE 3:**
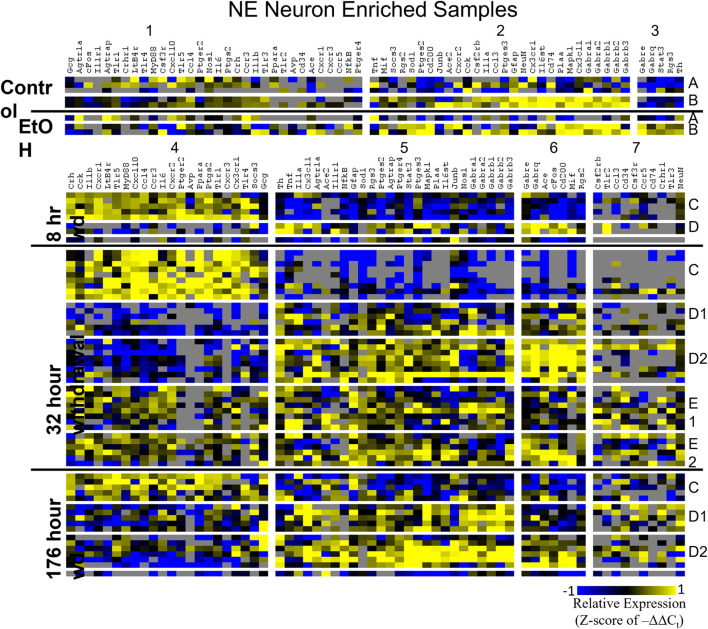
Heat Map of NE Neurons. Heat map displays cellular subphenotypes within NE neurons enriched samples through alcohol withdrawal time series. Rows represent 10-cell pooled samples with cellular subphenotype clusters labeled with uppercase letters. Columns represent the z-score of –ΔΔC_t_ gene expression values on a –1 to 1 color scale for that gene in that sample. Gene clusters are labeled by number.

**FIGURE 4:**
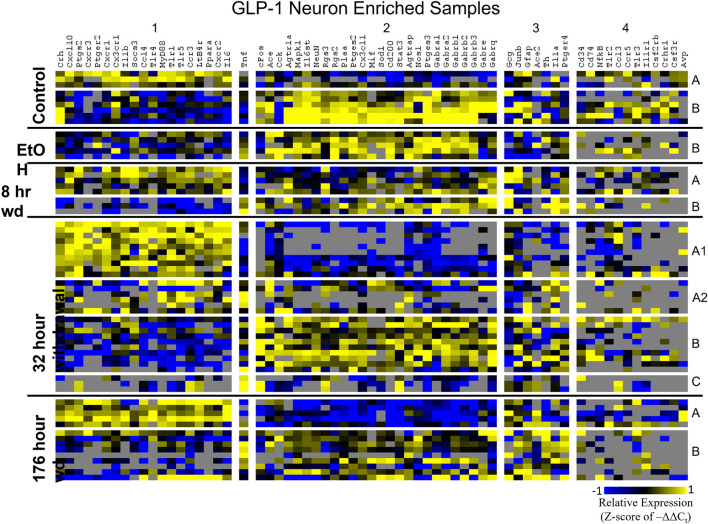
Heat Map of GLP-1 Neurons. Heat map displays cellular subphenotypes within GLP-1 neuron enriched samples through alcohol withdrawal time series. Rows represent 10-cell pooled samples with cellular subphenotype clusters labeled with uppercase letters. Columns represent the z-score of –ΔΔC_t_ gene expression values on a –1 to 1 color scale for that gene in that sample. Gene clusters are labeled by number.

**FIGURE 5:**
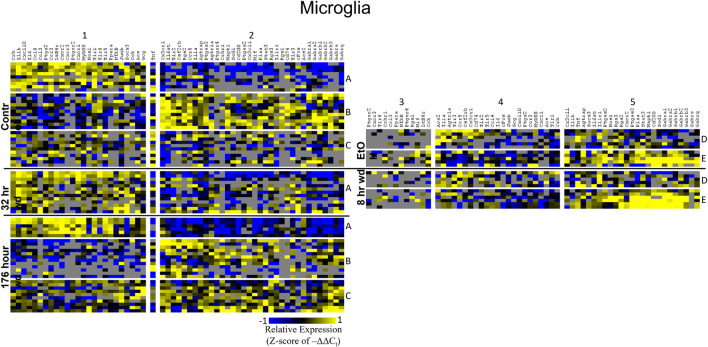
Heat Map of Microglia. Heat map displays microglia cellular subphenotypes through alcohol withdrawal time series. Rows represent 10-cell pooled samples with cellular subphenotype clusters labeled with uppercase letters. Columns represent the z-score of –ΔΔC_t_ gene expression values on a –1 to 1 color scale for that gene in that sample. Gene clusters are labeled by number.

Heat maps establishing well-defined data-driven cellular subphenotypes in neuronal samples and microglia were generated using Euclidean distance clustering of z-scores of the –ΔΔC_t_ values for each sample and gene in the dataset (Figures 3–5). Co-expression gene clusters are labeled with numbers and cellular subphenotype groupings are labeled with letters. GLP-1 neuron enriched samples had the same cellular subphenotypes with the same gene clusters across all treatments while NE neuron enriched samples and microglia had two co-expression configurations comprising the identified subphenotypes. The proportion of cells constituting a subphenotype in addition to gene cluster expression levels shifted with the treatment. GABA_*A*_R subunit genes clustered together in every configuration, and their expression was largely indicative of subphenotype groupings.

Two prominent subphenotypes, C and D, emerged in NE neuron enriched samples in withdrawal time points. Subphenotype C highly expressed gene cluster 4 which is rich in inflammatory ligands and receptors including *Crh, Il1b*, and *Ptgs2.* This was labeled the “inflammatory” gene cluster (see below). Subphenotype C suppressed gene cluster 5, which includes *Th* and GABA_*A*_R subunits, and 6. Subphenotype D, conversely, had the opposite expression pattern in these gene clusters. At 8 h wd, subphenotype C was predominant, but subphenotype D made up a higher proportion of cells at 32 and 176 h wd. Another subphenotype, E, emerged in the 32 h wd treatment that had moderately high expression of gene clusters 4 and 5. Subphenotypes D and E were further split into D1, D2, E1, and E2 based on medium or high expression, respectively, of gene cluster 6 which includes *Cd200*, *cFos*, and *Mif*.

Noradrenergic (NE) neuron enriched samples from control and EtOH treatments shared gene clusters distinct from the withdrawal treatments. GABA_*A*_R subunits were co-expressed consistent with all sampled cell types. GABA_*A*_R subunit genes showed high expression in subphenotype B in control and EtOH treatments, moderate expression in subphenotype D and E in 8 and 32 h wd treatments, and returned to high expression in 176 h wd subphenotype D. In subphenotype E, only found at the 32 h wd time point, all assayed genes were at least moderately expressed which may suggest that the regulatory mechanisms of gene expression that control this transcriptomic profile are activated at this phase of the withdrawal process. This observation requires further mechanistic study.

Co-expression genes clusters in GLP-1 neuron enriched samples were consistent throughout the time series. Subphenotype A highly expressed “inflammatory” gene cluster 1 rich in cytokine and chemokine ligands and receptors including *Crh*, *Il1b*, and *Ptgs2*, while suppressing “GABA_*A*_R” gene cluster 2. Subphenotype B had the opposite pattern. GLP-1 co-expression clusters 1 and 2 were surprisingly similar to NE co-expression clusters 4 and 5, again suggesting the mechanisms of regulatory constraint are shared between these phenotypes. Interestingly, GLP-1 subphenotype B emerged only in the EtOH treatment. At 8 h wd, subphenotypes A and B suppressed expression of their high-expressing gene clusters, 1 and 2, respectively, compared to control. At 32 and 176 h wd, subphenotype A gene cluster 1 was more highly expressed than in the control condition. Subphenotype B gene demonstrated a steady decrease in expression of gene cluster 2, and by the 176 h wd time had only moderate expression of “GABA_*A*_R” gene cluster 2. Concurrently, expression of gene cluster 3 for subphenotype B consistently increases throughout the withdrawal process and by 176 h wd is the most prominently upregulated gene cluster. *Tnf* did not group into any gene cluster and is isolated in the GLP-1 neuron enriched samples heat map to display this clearly (Figure 4).

Microglia shared co-expression clusters in subphenotypes A, B, and C for control, 32 h wd, and 176 h wd treatments. EtOH and 8 h wd treatments shared co-expression clusters in subphenotypes D and E. Subphenotype A was the exclusive expression pattern for the 32 h wd treatment which upregulated “inflammatory” gene cluster 1 including *Crh*, *Il1b*, and *Ptgs2*. This subphenotype A is most similar to the so-called M1 phenotype (Murray et al., 2014). At 176 h wd, subphenotype A made up a low proportion of samples while subphenotype C moderately expressed all genes assayed and more robustly than in the control treatment. *Tnf* did not group into a gene cluster for microglia either. High expression of GABA_*A*_Rs and a few other genes including *Sod1*, *Cd200*, *Mapk1*, and *Stat3* characterized Subphenotype E in the EtOH and 8 h wd treatments.

**FIGURE 6:**
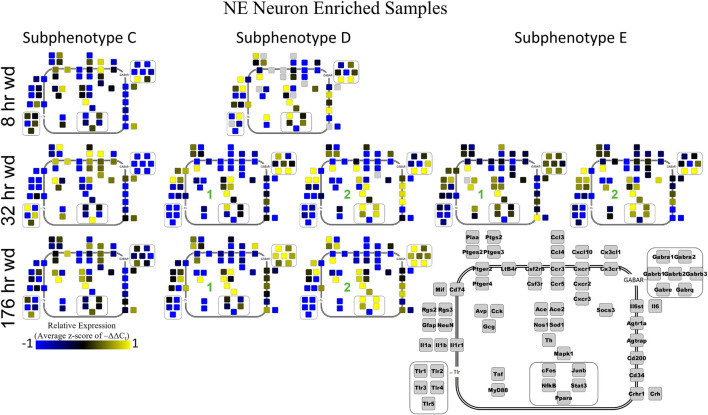
Subphenotype Gene Expression in Norepinephrine Neuron Enriched Samples. Cellular diagrams display boxes representing relative gene expression (average z-score of –ΔΔC_t_ values) of subphenotypes shown in prior heatmaps. Legend with gray boxes in lower right labels which boxes correspond to which gene. Box color represents expression (blue is low expression and yellow is high expression). The location of the box represents the localization or function of the protein product from that gene transcript. Green numbers indicate subgroups within subphenotypes. Groups A and B shown in Supplementary Figure 5.

**FIGURE 7:**
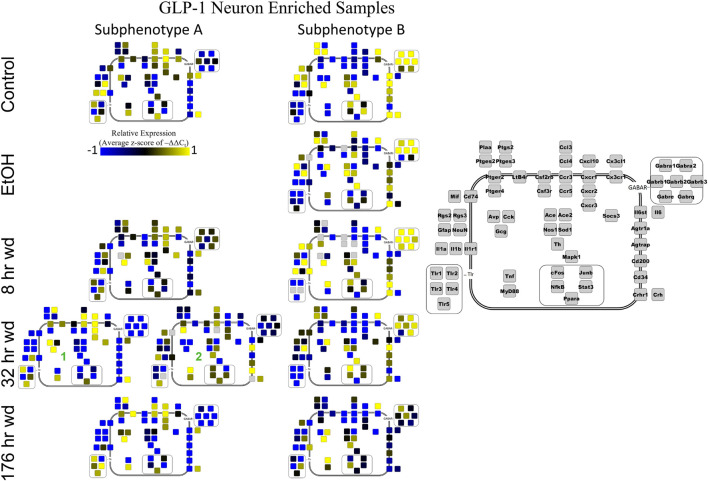
Subphenotype Gene Expression in GLP-1 Neuron Enriched Samples. Cellular diagrams display boxes representing relative gene expression (average z-score of –ΔΔC_t_ values) of subphenotypes shown in prior heatmaps. Legend with gray boxes in on right of figure labels which boxes correspond to which gene. Box color represents expression (blue is low expression and yellow is high expression). The location of the box represents the localization or function of the protein product from that gene transcript. Green numbers indicate subgroups within subphenotypes. Group C shown in Supplementary Figure 5.

**FIGURE 8:**
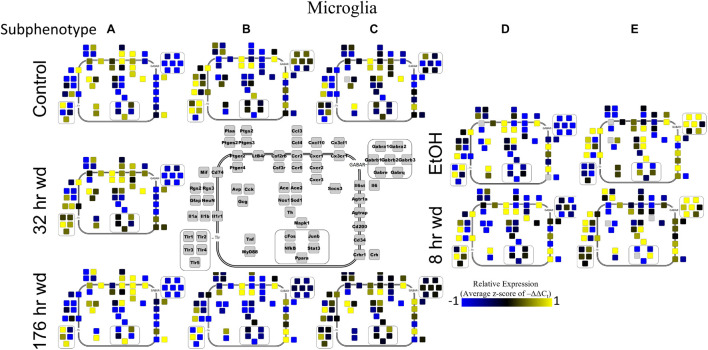
Subphenotype Gene Expression in Norepinephrine Neuron Enriched Samples. Cellular diagrams display boxes representing relative gene expression (average z-score of –ΔΔC_t_ values) of subphenotypes shown in prior heatmaps. Legend with gray boxes in center of figure labels which boxes correspond to which gene. Box color represents expression (blue is low expression and yellow is high expression). The location of the box represents the localization or function of the protein product from that gene transcript.

Next, single-cell samples were combined within their subphenotypes to yield an average value of expression for each gene within that subphenotype. This data is displayed in the cellular diagrams of Figures 6–8. Each box represents an assayed gene. Its color indicates the average z-score of –ΔΔC_t_ expression values. The location of this box in the cellular cartoon corresponds to the protein function of that gene. These diagrams provide a higher-level display of the functional state of the subphenotype at that time point and display the transcriptional dynamics occurring in each subphenotype in a readable way. In brief, Figure 6 shows a clear upregulation of GABA_*A*_R genes at 176 h wd as compared to 8 h wd in Group C and D of NE neuron enriched samples. Additionally, CD200 expression is one of the primary distinguishers of Group D1 vs. Group D2. Figure 7 displaying GLP-1 neuron enriched samples shows that GABA_*A*_R gene expression at 176 h wd is decreased in both subphenotypes. At the 32 h wd time point, *Cxcl10*, *Cxcr1*, *Cxcr2*, and *Cxcr3* expression distinguish Group A1 most prominently from Group A2. Microglia displayed in Figure 8 showed the most *Tnf* expression in groups D and E, subphenotypes only identified at the EtOH and 8 h wd treatments. *Cx3cr1*, a mircoglia gene prominently involved in neuronal adhesion, showed increased expression at the 176 h wd time point in all subphenotypes (Wolf et al., 2013). GLP-1 neuron enriched samples Group B had the most increased *Cx3cl1* expression possibly indicated this subphenotype interacts most with microglia at this time point.

## Discussion

Nucleus tractus solitarius (NTS) neurons regulate emotion, autonomic homeostasis, and stress responses. Multiple neuronal nuclei, ligands, receptors, and signaling dynamics are involved in these complex functions including NE, GLP-1, CRH, and GABA (Figures 1A,B). Moreover, local glial-neuronal paracrine signaling via inflammatory cytokines like tumor necrosis factor-alpha (TNF-α) also play a role. We microdissected single Th + neurons, Th- neurons, and microglia from the rat NTS as 10-cell pooled samples using LCM and measured their expression of 96 gene transcripts in an alcohol withdrawal time series (Supplementary Figure 1). Time points were chosen based on rat alcohol metabolism and withdrawal symptomatology (Hunter et al., 1975; Walker et al., 1975; Geisler et al., 1978; Macey et al., 1996). 8 h wd represents the start of acute AWS, 32 h wd represents the end of acute AWS, and 176 h wd represents a protracted withdrawal state. Physiological parameters and symptoms of withdrawal were not assessed. We found that neurons that stained Th + had significantly elevated *Th* expression and labeled these NE neuron enriched samples (Figure 1D). Th- neurons had significantly elevated expression of the GLP-1 precursor transcript *Gcg* and were labeled as neuronal samples enriched with GLP-1 + neurons. Likewise, CD11β + cells expressed the microglial markers *Cd34* and *Cx3cr1* at significantly elevated levels and were labeled microglia. In a dimension reduction analysis (LDA), these three cell types formed distinct clusters with microglia separating out from neurons along the x-axis and NE and GLP-1 neuron enriched samples separating along the y-axis suggesting these samples, are indeed, comprised of different cellular phenotypes (Figure 1E).

Further analysis of *Th* and *Gcg* expression showed an inverse relationship with respect to time point with *Gcg* expression demonstrating elevated expression levels only during withdrawal (Figures 2A,B). However, expression of these neurotransmitter precursor genes did not organize the other genes assayed into distinct subphenotypes correlated to their expression levels (Figures 2C,D). A data-driven approach to cellular subphenotype organization identified stark subphenotypes unique to each cell type likely with discrete functions (Figures 3–5). Strikingly, these subphenotypes shared similarities in their expression of their inflammatory gene clusters (Supplementary Table 4). Single-cell pooling may contribute to the observed heterogeneity of transcriptomic subphenotypes though single-neuron datasets also demonstrate high heterogeneity (Park et al., 2014; Bakken et al., 2018). Both of these variables likely contribute to the subphenotypes observed in this study.

Gene cluster 4 in NE neurons enriched samples, gene cluster 1 in GLP-1 neuron enriched samples, and gene cluster 1 in microglia samples constituted these “inflammatory” clusters (Supplementary Table 4). 18 genes were shared across all of these co-expression clusters and only 5 genes were unique to a single cluster suggesting similar mechanisms across cell types that regulate their expression. In NE enriched neuronal samples, subphenotype C highly expressed this inflammatory cluster while subphenotype E had moderate inflammatory co-expression cluster elevation. At 8 h wd, NE subphenotype C was 62.5% of the samples (5/8) and at 32 h wd C and E combined to 62.2% of the samples (23/37). By 176 h wd, subphenotype C was only 29% of NE neuron enriched samples (5/17). This may suggest that this subphenotype of NE neurons experiences a marked increase in inflammation during acute AWS, but that this subphenotype is not involved in protracted withdrawal symptoms such as low-grade anxiety (Breese and Knapp, 2016). This increase in local paracrine inflammation may increase the excitability for this NE neuron subphenotype (Schäfers and Sorkin, 2008). These expression data are consistent with clinical observations of hypersympathetic activity in acute, but not prolonged, AWS (Kosten and O’Connor, 2003). These observations inform future mechanistic approaches to confirm this hypothesis-generating dataset.

In GLP-1 neuron enriched samples, subphenotype A highly expressed the “inflammatory” gene cluster (gene cluster 1). The pattern of expression in this inflammatory subphenotype of GLP-1 neuron enriched samples (A) is similar to the inflammatory subphenotypes of NE neuron enriched samples (C and E). In samples enriched with GLP-1 neurons, subphenotype A makes up 33.3% of control samples (3/9), 0% of EtOH samples, 62.5% (5/8) of 8 h wd samples, and 55.2% (16/29) of 32 h wd samples. By 176 h wd, this inflammatory subphenotype has decreased back near control levels: 35.7% (5/14) of GLP-1 neurons.

Surprisingly, microglia demonstrated a similar pattern. Microglia subphenotype A also highly expressed the inflammatory gene cluster (cluster 1). High gene expression in this cluster is indicative of M1 microglia phenotypes as this cluster includes the M1 markers Il1β, Il6, Nos1, Ptgs2, and TLRs 1,4, and 5 (Martinez and Gordon, 2014). This phenotype made up 29.0% (9/31) of the control samples, 100% (13/13) of the 32 h wd samples, and only 21.4% of the 176 h wd samples. Of note, all NTS microglia sampled at the 32 h wd time points demonstrated an M1 phenotype which may indicate neuroinflammation in the NTS during the acute phase of AWS. Conversely, we observed fewer M1-like microglia at 176 h wd compared to control samples which is unexpected based on our previous work on alcohol withdrawal in the amygdala (Freeman et al., 2012a, b, 2013). We expected neuroinflammatory markers to be increased at the 176 h wd time point, especially in microglia, but these data suggest that the NTS experiences inflammation during acute withdrawal only (8 h and 32 h time points) and recovers by the 176 h time point. Indeed, we observe less neuroinflammation at 176 h wd in all three cell types assayed. One speculatory explanation for this observation is that compensatory endogenous anti-inflammatory signaling may be happening at this timepoint, though we cannot substantiate this claim with the genes measured in this study.

The 176 h wd time point is meant to measure long term changes in gene expression that occur in protracted withdrawal. At this time point, some similarities across cell types were observed in the subphenotypes that highly expressed GABAR subunits as was observed at other time points. NE neuron enriched sample cluster 5, GLP-1 neuron enriched sample cluster 2 and microglia cluster 2 contained the majority of the GABAR subunit genes, and the makeup of this “GABAR” co-expression cluster was not as consistent as the inflammatory cluster across cell types—16 genes are shared across all cell types and 16 genes are unique to a single cell type within its respective GABAR cluster (Supplementary Table 4). GLP-1 neuron enriched samples in subphenotype B upregulates this co-expression cluster in the control treatment, but the relative level of expression of this cluster decreases throughout the time series within this subphenotype (Figure 4). At the 176 h wd time point, this GABAR cluster is only moderately expressed which may suggest long term changes to this neuronal subphenotype following alcohol dependence and withdrawal. The decrease in expression of inhibitory GABAR gene transcripts, along with the concurrent upregulation of co-expression cluster 3, which contains *Gcg*, may suggest that this GLP-1 enriched sample subphenotype increases its GLP-1 neurotransmission in protracted withdrawal. Literature indicates that GLP-1 signaling from the NTS to the amygdala and other nuclei is anxiogenic (Rinaman, 1999). Taken together, these data are consistent with this GLP-1 enriched neuronal subphenotype not playing a role in the acute withdrawal process characterized by inflammation, but rather experiencing GABAR subunit downregulation over a longer process potentially leading to increased anxiety and susceptibility to stress in protracted AWS. However, this is a purely speculative conjecture.

Microglia also showed elevated GABAR expression at the 176 h wd time point, but the pattern of increased GABAR expression was unexpected. Control microglia in subphenotype C show moderate expression of both cluster 1 (inflammatory) and cluster 2 (GABA_*A*_R) (Figure 5). Expression of both clusters increase at the 176 h wd time point. This may suggest elevated inflammation, but not by distinct M1 phenotype microglia (subphenotype A), and also elevated GABAR expression. These observations are best visualized in Figure 8. Of note, there are many genes in microglia cluster 2 that are not GABAR subunits. Moreover, microglial *Tnf* expression was significantly elevated in control, EtOH and 8 h wd treatments compared to 176 h wd independent of subphenotype (Supplementary Table 3). Indeed, *Tnf* expression by microglia did not fit neatly into a gene cluster. Cluster C has some cells that demonstrate high *Tnf* expression in both control and 176 h wd, where Cluster A showed a decrease in *Tnf* expression between these two time points. Cluster B, conversely, increased its expression of *Tnf* from control to 176 h wd. This apparent absence of a pattern in microglia *Tnf* expression suggests that in microglia this gene that is central to neuroinflammation is constrained by a mechanism that is independent of the other genes measured in this study. Further, the decrease in overall microglia *Tnf* expression at 176 h wd as measured by an average of –ΔΔC_t_ values and two-tailed heteroscedastic *t*-tests may be misleading. A single-cell analysis reveals that overall expression may not be the best indicator of inflammation. Rather, shifts in subphenotype proportion, and the number of cells showing a moderately increased *Tnf* expression, as seen in subphenotype C, may have more of a physiologic impact than total gene expression levels.

Cell diagrams in Figures 6–8 average the expression of a gene within a subphenotype designated by color and display that color in a location on the diagram that corresponds to the protein function. This method of data presentation allows for analysis of receptor-ligand interactions within and between subphenotypes. For example, Figure 6 displaying NE neuron enriched samples shows that at 32 h wd, subphenotype C experiences an increase in expression of ligand-receptor pair *Ccl-Ccr* and *Cxcl10-Cxcr*. This may indicate that CCL-CCR and CXCL10-CXCR signaling is elevated at this timepoint in AWS. Figure 6 also provides clarity in subphenotype D upregulation of *Mapk1* at 176 h wd which may suggest long term transcription is altered during protracted withdrawal in this subphenotype. Moreover, transcription factor genes *cFos*, *Junb*, *NfkB*, and *Stat3* have increased expression in subphenotype D2 which is consistent with this subset of NE neuron enriched samples experiencing long-term changes in transcription following alcohol withdrawal. Microglia in subphenotype C upregulate *IL1a*, *IL1b*, and *IL1r1* at 176 h wd in subphenotype C as compared to control, while subphenotype B downregulate these genes at 176 h wd compared to control (Figure 8). This dynamic may suggest that subphenotype B provides an anti-inflammatory function that is most active in protracted withdrawal. Similarly, it may suggest that microglia subphenotype C, identified here as a microglia subset that can function in a multitude of processes whether inflammatory or anti-inflammatory based on their lack of a clear co-expression module pattern in control, is pushed toward an inflammatory state in protracted withdrawal.

This dataset has allowed the identification of cellular subphenotypes and their gene expression dynamics in alcohol withdrawal through time. The fusion of single cells into 10-cell pools can result in some obfuscation of phenotypic dynamics; The details of which can be resolved at a higher resolution. Nevertheless, this analysis has revealed valuable observations in both neurotransmission signaling and local paracrine signaling processes that aid in hypothesis-generation while relating to what is observed clinically in the context of what is already established about such neurotransmission. The dataset is unique in that microfluid RT-qPCR, a method lower in throughput but more reliable than RNA-seq (SEQC/MAQC-III Consortium, 2014), is combined with anatomic and staining specificity using LCM for single-cell selection in a time series. This allows for analysis of complex signaling dynamics at multiple levels, and the influence of such signaling dynamics on both acute AWS and protracted withdrawal based on the clinical symptoms at that time point. However, this hypothesis-generating study from which functional correlates cannot be determined.

The major weakness of this study is the number of animals assayed. Ten rats total were assayed and single cells were collected from a single animal from some conditions (control neurons, chronic ethanol, 8 h wd, 32 h wd microglia). This design was due to both cost, and previous studies from our group that consistently demonstrate that single-cells within an animal have as much transcriptional heterogeneity, or variance, as between animals (Park et al., 2014, 2016; O’Sullivan et al., 2019). This is observed in both true single-cell and pooled sample studies. In this dataset of 10-cell pooled samples, this phenomenon was also observed suggesting that a single animal that is heavily sampled does not bias the dataset and contains as much variability as multi-animal single-cell or pooled single-cell studies (Supplementary Figure 2). In addition, we did not measure alcohol withdrawal symptomatology or animal weight here as these features of alcohol withdrawal from this protocol have been well-characterized elsewhere (Hunter et al., 1975; Walker et al., 1975).

We have collected the data, validated the accuracy of the dataset, and identified cellular subphenotypes and their major signaling dynamics. However, signaling dynamics measured in our dataset can be further investigated and may identify clinical targets to treat acute or protracted AWS and potentially alcohol dependence itself. Future studies analyzing these signaling dynamics with the addition of female rats that also include other brain cell types such as astrocytes and endothelial cells are needed to further understand the underlying pathophysiology of AWS and dependence.

Lastly, these findings are consistent with our hypothesis that neuroinflammation in the visceral-emotional neuraxis contributes to antireward which motivates alcohol, and opioid, dependence (Figures 1A,B and Supplementary Figure 7) (O’Sullivan and Schwaber, 2021). The work of others supports this conjecture as well (Koob and Le Moal, 2008a; de Timary et al., 2015; Skosnik and Cortes-Briones, 2016; Meckel and Kiraly, 2019; Carbia et al., 2021; Wang et al., 2021). In brief, the hypothesis suggests that neuroinflammation in the NTS and amygdala stimulates antireward which contributes to negative reinforcement. This study not only provides evidence of neuroinflammation in the NTS in acute and protracted alcohol withdrawal, but also an understanding of the emergence of this neuroinflammation and its relation to neurotransmission and AWS. Improved understanding of such processes in alcohol withdrawal lends insights into targets that may mitigate inflammation, decrease antireward in AWS, and treat substance dependence.

## Materials and Methods

### Animals

Approval of protocols was given by Institutional Animal Care and Use Committee of Thomas Jefferson University. The study was carried out in compliance with ARRIVE guidelines and in accordance with all relevant guidelines and regulations. Ten young, male, Sprague Dawley rats (35–45 grams) ordered from Harlan Laboratory were housed individually in the Thomas Jefferson University Alcohol Research Center Animal Core Facility. Standard chow and water were given until rats weighed 120 grams. Rats were then fed an alcohol-free, maltose-dextrin substituted, Lieber-DeCarli liquid diet (bioServe, Frenchtown, NJ) for three days (Lieber and Decarli, 1974). Animals were then assigned to five treatment groups: control, chronic alcohol exposure (EtOH), 8-h wd, 32 h wd, or 176 h wd (Supplementary Figure 1). Animals received eight months of continuous ethanol (36% of calories as ethanol) or control diet *ad libitum* (Lieber and Decarli, 1974). Control diet animals received a quantity of the liquid diet that equaled the caloric intake of the matched alcohol-fed animal 24-h prior. No other water or chow was provided ensuring all fluid and nutrient intake came from alcohol diet. Withdrawal animals were withdrawn such that sacrifice by rapid decapitation was at the same circadian time for all conditions. During withdrawal, animals received the control diet *ad libitum*. Previous studies have shown blood alcohol concentration to average between 20 and 30 mM with daily intake of 12–16 g/kg and linear metabolism for Lieber-DeCarli protocols (Wilson et al., 1986; Lieber and Decarli, 1994). Previous studies from our facility had similar findings (Freeman et al., 2012b). Following dependence, which can occur after just 10 days of this protocol (Wilson et al., 1986), significant AWS symptoms begin at 4 h abstinence and resolve around 72 h as we and others have noted (Hunter et al., 1975; Walker et al., 1975; Macey et al., 1996; Freeman et al., 2012b, 2013). This timeline informed the time points for this study. Single-cell gene expression demonstrated similar or greater variance within an animal as that between animals (Supplementary Figure 2). That is, single-cell gene expression did not differ substantially between animals suggesting one animal heavily sampled is statistically similar to multiple animals sampled moderately.

### Rapid Decapitation, Fast Staining Protocol, Laser Capture Microdissection

Dissected brainstems were frozen in Optimal Cutting Temperature (O.C.T.) following rapid decapitation for cryostat sectioning and stored at -80°C for nucleic acid preservation. An in-house rapid immunofluorescent staining protocol developed to preserve nucleic acid integrity was used to visualize cell types for single-cell LCM as explained elsewhere (Supplementary Figure 1B) (Park et al., 2016). Briefly, 10 μm thick brain sections were thaw-mounted onto glass slides. 30 s of 75% ethanol fixed sliced tissue. 30 s of 2% BSA (Sigma-Aldrich) in phosphate-buffered saline (PBS) was used for blocking. Tissue was then stained with 3% of anti-NeuN antibody (EMD Millipore, MAB377) or anti-Cd11β antibody (Genway Biotech, CCEC48) for neuron and microglia primary labeling, respectively. 3% anti-tyrosine hydroxylase (Th) antibody (Abcam, ab112) was also used on NeuN stained slides for noradrenergic neuron labeling. Secondary antibodies (ratio 1:200) goat anti-mouse Alexa Fluor-555 and donkey anti-rabbit Alexa Fluor-488 were used for cell type and Th fluorescence, respectively. DAPI (1:10000) stained cell nuclei. PBS wash ensued, along with a standard alcohol dehydration protocol of time series baths (30 s 75% ethanol, 30 s 95% ethanol, 30 s 100% ethanol, 30 s 100% ethanol, 60 s xylenes, 4 min xylenes) and 5 min in desiccator before LCM.

### Single Cell Sampling and High-Throughput RT-qPCR

3230 single brain cells, 950 Th + neurons, 1030 Th− neurons, and 1250 microglia, were collected from the NTS using LCM. Cells were grouped into 10-cell pools comprising 323 total samples analyzed. This pooling of cells increases the number of samples analyzed by the microfluidic RT-qPCR platform. cDNA from mRNA transcripts was generated by reverse transcription (SuperScript^TM^ VILO^TM^ cDNA Synthesis Kit; ThermoFisher). TaqMan PreAmp Master Mix was used for pre-amplification of cDNA (22 cycles) with forward and reverse PCR primers (96 pairs). The Biomark microfluidic qPCR platform (Fluidigm©) was used to measure expression levels of 96 genes. Four batches of probe-based qPCR measured the previously amplified 96 cDNA transcripts. Supplementary Table 1 lists primers used. Primer amplicon validation was performed on agarose gel electrophoresis. Following strict quality control protocols, a total of 229 10-cell pooled samples (70 NE neuron samples, 65 GLP-1 neuron samples, and 94 microglial samples) and 65 gene transcripts were used for data analysis.

The four microfluidic RT-qPCR batches run for this study were assessed for intra- and inter-batch experimental quality (Supplementary Figures 3, 4). Technical replicates assessing intra-batch quality demonstrated high similarity with r values listed (Supplementary Figure 3). Inter-batch replicates demonstrated high batch similarity, though batch 4 sample 40 showed contamination (Supplementary Figure 4). A dilution series using standard rat brain RNA was also included in each batch for quantitative analysis (Supplementary Table 2); However, the data normalization method explained below calculated relative expression and was used for all analysis in this study.

### Data Normalization

A two-step median-centering –ΔΔC_t_ method was used for expression level normalization was explained elsewhere (Achanta et al., 2018). Briefly, a raw C_t_ value was obtained for each gene and sample. Each individual C_t_ value was normalized to the overall sample median [(Median sample expression) – C_t gene_ = –ΔC_t sample_]. The newly obtained –ΔC_t_ values were then median-centered to the gene across all samples [–ΔC_t sample_ – (Across sample –ΔC_t_ median) = –ΔΔC_t gene_]. This yields a –ΔΔC_t_ value for each measurement allowing comparison of relative gene expression values across treatment groups and batches. This analysis was carried out in R version 3.5.2. The raw C_t_ values are listed in Supplementary Table 2 without gene quality control. The normalized dataset with quality-control that was used for all analysis is also displayed in Supplementary Table 2.

## Data Availability Statement

The original contributions presented in the study are included in the article/Supplementary Material, further inquiries can be directed to the corresponding author.

## Ethics Statement

The animal study was reviewed and approved by Institutional Animal Care and Use Committee of Thomas Jefferson University.

## Author Contributions

SO’S performed microfluidic qPCR, data analysis, figure generation, and writing of manuscript. DM-C collected single-cell samples under the guidance of JP. JP also designed the experiments. RV and JS were involved with figure design and editing. All authors discussed the results and commented on the manuscript.

## Conflict of Interest

The authors declare that the research was conducted in the absence of any commercial or financial relationships that could be construed as a potential conflict of interest.

## Publisher’s Note

All claims expressed in this article are solely those of the authors and do not necessarily represent those of their affiliated organizations, or those of the publisher, the editors and the reviewers. Any product that may be evaluated in this article, or claim that may be made by its manufacturer, is not guaranteed or endorsed by the publisher.

## Supplementary Material

Supplementary Figure 1 Experimental design and single-cell selection. **(A)** Rat triplets were randomly assigned one of five treatments studied. **(B)** Single-cell selection and measure of transcription.

Supplementary Figure 2 Density plots of normalized data across all genes. **(A)** Three separate rats provided single-cell samples for 32 h wd neurons. Gene expression variance between animals was similar or less than gene expression variance within an animal. (All 32 h wd neurons N = 3093, variance = 4.16; Animal 6 N = 1743, variance = 2.88; Animal 9 N = 730, variance = 5.76; Animal 10 N = 620, variance = 5.58). **(B)** (All 32 h wd NE neurons N = 1815, variance = 3.96; Animal 6 N = 1112, variance = 3.04; Animal 9 N = 391, variance = 4.24, Animal 10 N = 312, variance = 6.63). **(C)** (All 32 h wd GLP-1 neurons N = 1278, variance = 4.44; Animal 6 N = 631, variance = 2.60, Animal 9 N = 339, variance = 7.39, Animal 10 N = 308, variance = 4.52). **(D)** Two separate rats provided single-cell samples for control microglia. Gene expression variance between animals was similar or less than gene expression variance within an animal (All Control Microglia N = 1451, variance = 3.97; Animal 2 N = 958, variance = 3.40; Animal = 4 N = 493, variance = 5.01).

Supplementary Figure 3 Technical replicate plots of raw Ct values.

Supplementary Figure 4 Inter-batch replicate plots of raw Ct values.

Supplementary Figure 5 Inter-batch replicate plots of raw Ct values.

Supplementary Figure 6 Suphenotype gene expression in NE and GLP-1 neurons. Cellular cartoons display boxes representing relative gene expression (average z-score of −ΔΔCt values) of subphenotypes shown in prior heatmaps. Legend on right labels which boxes correspond to which gene and the color that represents expression (blue is low expression and yellow is high expression). The location of the box represents the localization or function of the protein product from that gene transcript. Legend is shown in gray boxes with labels.

Supplementary Figure 7 Schematic of opponent-process model of addiction. Originally published in O’Sullivan and Schwaber (2021). **(A)** Alcohol and/or opioid exposure has two actions: Stimulate reward, via the mesolimbic dopamine pathway, and inhibit antireward. These actions motivate substance use via positive reinforcement. **(B)** Alcohol and/or opioid withdrawal has two action. Inhibit reward, by inhibiting the mesolimbic dopamine pathway (not shown), and stimulate antireward. This study proposes that visceral-emotional neuroinflammation is an endpoint in antireward stimulation, though this hypothesis warrants further testing. These actions, whatever the mechanism, motivate substance dependence via negative reinforcement.

## References

[B1] Achanta S. Verma A. Srivastava A. Nilakantan H. Hoek J. B. Vadigepalli R. (2018). Single cell gene expression analysis identifies chronic alcohol-mediated shift in hepatocyte molecular states after partial hepatectomy. *Gene Expr.* 19 97–119. 10.3727/105221618X15361728786767 30189915 PMC6466177

[B2] Aimino M. Coker C. Silberman Y. (2018). Acute ethanol modulation of neurocircuit function in the nucleus of the tractus solitarius. *Brain Res. Bull.* 138 5–11. 10.1016/j.brainresbull.2017.07.019 28760662 PMC5788745

[B3] Armstrong D. M. Ross C. A. Pickel V. M. Joh T. H. Reis D. J. (1982). Distribution of dopamine-, noradrenaline-, and adrenaline-containing cell bodies in the rat medulla oblongata: demonstrated by the immunocytochemical localization of catecholamine biosynthetic enzymes. *J. Comp. Neurol.* 212 173–187. 10.1002/cne.902120207 6142061

[B4] Baker T. B. Piper M. E. McCarthy D. E. Majeskie M. R. Fiore M. C. (2004). Addiction Motivation Reformulated: an Affective Processing Model of Negative Reinforcement. *Psychol. Rev.* 111 33–51. 10.1037/0033-295X.111.1.33 14756584

[B5] Bakken T. E. Hodge R. D. Miller J. A. Yao Z. Nguyen T. N. Aevermann B. (2018). Single-nucleus and single-cell transcriptomes compared in matched cortical cell types. *PLoS One* 13:e0209648. 10.1371/journal.pone.0209648 30586455 PMC6306246

[B6] Bär K. J. Boettger M. K. Boettger S. Grotelüschen M. Neubauer R. Jochum T. (2006). Reduced baroreflex sensitivity in acute alcohol withdrawal syndrome and in abstained alcoholics. *Drug Alcohol. Depend.* 85 66–74. 10.1016/j.drugalcdep.2006.03.014 16650658

[B7] Breese G. R. Knapp D. J. (2016). Persistent adaptation by chronic alcohol is facilitated by neuroimmune activation linked to stress and CRF. *Alcohol* 52 9–23. 10.1016/j.alcohol.2016.01.005 27139233 PMC4855305

[B8] Carbia C. Lannoy S. Maurage P. López-Caneda E. O’Riordan K. J. Dinan T. G. (2021). A biological framework for emotional dysregulation in alcohol misuse: from gut to brain. *Mol. Psychiatry* 26 1098–1118. 10.1038/s41380-020-00970-6 33288871

[B9] Covarrubias M. Y. Khan R. L. Vadigepalli R. Hoek J. B. Schwaber J. S. (2005). Chronic alcohol exposure alters transcription broadly in a key integrative brain nucleus for homeostasis: the nucleus tractus solitarius. *Physiol. Genomics* 24 45–58. 10.1152/physiolgenomics.00184.2005 16189278

[B10] de Timary P. Leclercq S. Stärkel P. Delzenne N. (2015). A dysbiotic subpopulation of alcohol-dependent subjects. *Gut Microbes* 6 388–391. 10.1080/19490976.2015.1107696 26727422 PMC4826101

[B11] DeCicco D. Zhu H. Brureau A. Schwaber J. S. Vadigepalli R. (2015). MicroRNA network changes in the brain stem underlie the development of hypertension. *Physiol. Genomics* 47 388–399. 10.1152/physiolgenomics.00047.2015 26126791 PMC4556940

[B12] Ferry B. Roozendaal B. McGaugh J. L. (1999). Role of norepinephrine in mediating stress hormone regulation of long-term memory storage: a critical involvement of the amygdala. *Biol. Psychiatry* 46 1140–1152. 10.1016/s0006-3223(99)00157-2 10560021

[B13] Freeman K. Brureau A. Vadigepalli R. Staehle M. M. Brureau M. M. Gonye G. (2012a). Temporal changes in innate immune signals in a rat model of alcohol withdrawal in emotional and cardiorespiratory homeostatic nuclei. *J. Neuroinflamm.* 9:97. 10.1186/1742-2094-9-97 22626265 PMC3411448

[B14] Freeman K. Staehle M. M. Gümüş Z. H. Vadigepalli R. Gonye G. E. Nichols C. N. (2012b). Rapid temporal changes in the expression of a set of neuromodulatory genes during alcohol withdrawal in the dorsal vagal complex: molecular evidence of homeostatic disturbance. *Alcohol. Clin. Exp. Res.* 36 1688–1700. 10.1111/j.1530-0277.2012.01791.x 22486438 PMC4419739

[B15] Freeman K. Staehle M. M. Vadigepalli R. Gonye G. E. Ogunnaike B. A. Hoek J. B. (2013). Coordinated dynamic gene expression changes in the central nucleus of the amygdala during alcohol withdrawal. *Alcohol. Clin. Exp. Res.* 37 E88–E100. 10.1111/j.1530-0277.2012.01910.x 22827539 PMC4408903

[B16] Geisler R. F. Hunter B. E. Walker D. W. (1978). Ethanol dependence in the rat: temporal changes in neuroexcitability following withdrawal. *Psychopharmacology* 56 287–292. 10.1007/BF00432851 418436

[B17] Grodin E. Bujarski B. Burnette E. Nieto S. Lim A. Lin J. (2021). Ibudilast, a neuroimmune modulator, reduces heavy drinking and alcohol cue-elicited neural activation: a randomized trial. *Transl. Psychiatry* 11:355. 10.1038/s41398-021-01478-5 34120149 PMC8197758

[B18] Gu G. Roland B. Tomaselli K. Dolman C. S. Lowe C. Heilig J. S. (2013). Glucagon-like peptide-1 in the rat brain: distribution of expression and functional implication. *J. Comp. Neurol.* 521 2235–2261. 23238833 10.1002/cne.23282

[B19] Han V. K. Hynes M. A. Jin C. Towle A. C. Lauder J. M. Lund P. K. (1986). Cellular localization of proglucagon/glucagon-like peptide I messenger RNAs in rat brain. *J. Neurosci. Res.* 16 97–107. 10.1002/jnr.490160110 2427741

[B20] Herman J. P. (2012). Neural pathways of stress integration relevance to alcohol abuse. *Curr. Rev.* 34 441–447. 10.35946/arcr.v34.4.08 PMC3860392 23584110

[B21] Holt M. K. Trapp S. (2016). The physiological role of the brain GLP-1 system in stress. *Cogent Biol.* 2:1229086. 10.1080/23312025.2016.1229086 27722184 PMC5043148

[B22] Hunter B. E. Riley J. N. Walker D. W. Freund G. (1975). Ethanol dependence in the rat: a parametric analysis. *Pharmacol. Biochem. Behav.* 3 619–629. 10.1016/0091-3057(75)90183-5 1237896

[B23] Jerlhag E. (2020). Alcohol-mediated behaviours and the gut-brain axis; with focus on glucagon-like peptide-1. *Brain Res.* 1727:146562. 10.1016/j.brainres.2019.146562 31759971

[B24] Kalia M. Fuxe K. Goldstein M. (1985). Rat medulla oblongata. II. Dopaminergic, noradrenergic (A1 and A2) and adrenergic neurons, nerve fibers, and presumptive terminal processes. *J. Comp. Neurol.* 233 308–332. 10.1002/cne.902330303 2858497

[B25] King A. C. Errico A. L. Parsons O. A. Lovallo W. R. (1991). Blood Pressure Dysregulation Associated with Alcohol Withdrawal. *Alcohol. Clin. Exp. Res.* 15 478–482. 1877732 10.1111/j.1530-0277.1991.tb00546.x

[B26] Koob G. F. (2009). Brain stress systems in the amygdala and addiction. *Brain Res.* 1293 61–75. 19332030 10.1016/j.brainres.2009.03.038 PMC2774745

[B27] Koob G. F. (2014). Neurocircuitry of alcohol addiction: synthesis from animal models. *Handb. Clin. Neurol.* 125 33–54. 10.1016/b978-0-444-62619-6.00003-3 25307567

[B28] Koob G. F. Le Moal M. (2001). Drug Addiction, Dysregulation of Reward, and Allostasis. *Neuropsychopharmacology* 24 97–129. 11120394 10.1016/S0893-133X(00)00195-0

[B29] Koob G. F. Le Moal M. (2008a). Addiction and the Brain Antireward System. *Annu. Rev. Psychol.* 59 29–53. 10.1146/annurev.psych.59.103006.093548 18154498

[B30] Koob G. F. Le Moal M. (2008b). Review. Neurobiological mechanisms for opponent motivational processes in addiction. *Philos. Trans. R. Soc. Lond. B. Biol. Sci.* 363 3113–3123. 10.1098/rstb.2008.0094 18653439 PMC2607326

[B31] Kosten T. R. O’Connor P. G. (2003). Management of Drug and Alcohol Withdrawal. *N. Engl. J. Med.* 348 1786–1795. 12724485 10.1056/NEJMra020617

[B32] Larsen P. J. Tang-Christensen M. Holst J. J. Orskov C. (1997). Distribution of glucagon-like peptide-1 and other preproglucagon-derived peptides in the rat hypothalamus and brainstem. *Neuroscience* 77 257–270. 10.1016/s0306-4522(96)00434-4 9044391

[B33] Lieber C. S. Decarli L. M. (1974). An experimental model of alcohol feeding and liver injury in the baboon. *J. Med. Primatol.* 3 153–163. 10.1159/000459999 4214518

[B34] Lieber C. S. Decarli L. M. (1994). Animal models of chronic ethanol toxicity. *Methods Enzymol.* 233 585–594. 10.1016/S0076-6879(94)33061-1 8015491

[B35] Macey D. J. Schulteis G. Heinrichs S. C. Koob G. F. (1996). Time-dependent quantifiable withdrawal from ethanol in the rat: Effect of method of dependence induction. *Alcohol* 13 163–170. 10.1016/0741-8329(95)02030-6 8814651

[B36] Maniscalco J. W. Rinaman L. (2018). Vagal Interoceptive Modulation of Motivated Behavior. *Physiology* 33 151–167. 10.1152/physiol.00036.2017 29412062 PMC5899236

[B37] Martinez F. O. Gordon S. (2014). The M1 and M2 paradigm of macrophage activation: time for reassessment. *F1000Prime Rep.* 6:13. 10.12703/P6-13 24669294 PMC3944738

[B38] McBride W. J. Kimpel M. W. Schultz J. A. McClintick J. N. Edenberg H. J. Bell R. L. (2010). Changes in gene expression in regions of the extended amygdala of alcohol-preferring rats after binge-like alcohol drinking. *Alcohol* 44 171–183. 10.1016/j.alcohol.2009.12.001 20116196 PMC2831121

[B39] McDonald M. Hoek J. Ogunnaike B. Schwaber J. (2008). Behavioral and neurobiological changes within a period of heightened susceptibility to voluntary alcohol withdrawal. *FASEB J. Federat. Am. Soc. Exp. Biol.* 22, 946.7–946.7. 10.1096/fasebj.22.1_supplement.946.7

[B40] Meckel K. R. Kiraly D. D. (2019). A potential role for the gut microbiome in substance use disorders. *Psychopharmacology vol.* 236 1513–1530. 10.1007/s00213-019-05232-0 PMC6599482 30982128

[B41] Murray P. J. Allen J. E. Biswas S. K. Fisher E. A. Gilroy D. W. Goerdt S. (2014). Macrophage Activation and Polarization: nomenclature and Experimental Guidelines. *Immunity* 41 14–20. 10.1016/j.immuni.2014.06.008 25035950 PMC4123412

[B42] O’Sullivan S. J. Schwaber J. S. (2021). Similarities in alcohol and opioid withdrawal syndromes suggest common negative reinforcement mechanisms involving the interoceptive antireward pathway. *Neurosci. Biobehav. Rev.* 125 355–364. 10.1016/j.neubiorev.2021.02.033 33647322 PMC8555739

[B43] O’Sullivan S. J. Reyes B. A. S. Vadigepalli R. Van Bockstaele E. J. Schwaber J. S. (2020). Combining laser capture microdissection and microfluidic qpcr to analyze transcriptional profiles of single cells: a systems biology approach to opioid dependence. *J. Vis. Exp.* 2020:e60612. 10.3791/60612 32202523 PMC8015684

[B44] O’Sullivan S. J. Malahias E. Park J. Srivastava A. Reyes B. A. S. Gorky J. (2019). Single-Cell Glia and Neuron Gene Expression in the Central Amygdala in Opioid Withdrawal Suggests Inflammation With Correlated Gut Dysbiosis. *Front. Neurosci.* 13:665. 10.3389/fnins.2019.00665 31333398 PMC6619439

[B45] Park J. Anthony B. Kate K. Alexandria S. Sonali G. Babatunde O. (2014). Inputs drive cell phenotype variability. *Genome Res.* 24 930–941. 10.1101/gr.161802.113 24671852 PMC4032857

[B46] Park J. Haisun Z. Sean O. S. Ogunnaike B. A. Weaver D. R. Schwaber J. S. (2016). Single-Cell Transcriptional Analysis Reveals Novel Neuronal Phenotypes and Interaction Networks Involved in the Central Circadian Clock. *Front. Neurosci.* 10:481. 10.3389/fnins.2016.00481 27826225 PMC5079116

[B47] Phelps E. A. LeDoux J. E. (2005). Contributions of the Amygdala to Emotion Processing: from Animal Models to Human Behavior. *Neuron* 48 175–187. 16242399 10.1016/j.neuron.2005.09.025

[B48] Ray L. A. Bujarski S. Shoptaw S. Roche D. J. Heinzerling K. Miotto K. (2017). Development of the Neuroimmune Modulator Ibudilast for the Treatment of Alcoholism: a Randomized, Placebo-Controlled, Human Laboratory Trial. *Neuropsychopharmacology* 42 1776–1788. 10.1038/npp.2017.10 28091532 PMC5520778

[B49] Reiner P. Vincent S. (1986). The distribution of tyrosine hydroxylase, dopamine-beta-hydroxylase, and phenylethanolamine-N-methyltransferase immunoreactive neurons in the feline medulla oblongata. *J. Comp. Neurol.* 248 518–531. 2873156 10.1002/cne.902480405

[B50] Retson T. A. Sterling R. C. Van Bockstaele E. J. (2015). Alcohol-induced dysregulation of stress-related circuitry: The search for novel targets and implications for interventions across the sexes. *Prog. Neuropsychopharmacol. Biol. Psychiatry* 10.1016/j.pnpbp.2015.05.009 26006055 PMC4656147

[B51] Rinaman L. (1999). Interoceptive stress activates glucagon-like peptide-1 neurons that project to the hypothalamus. *Am. J. Physiol.* 277 R582–R590. 10444567 10.1152/ajpregu.1999.277.2.R582

[B52] Roberto M. Kirson D. Khom S. (2020). The Role of the Central Amygdala in Alcohol Dependence. *Cold Spring Harb. Perspect. Med.* 11:a039339. 10.1101/cshperspect.a039339 31988201 PMC7382982

[B53] Schäfers M. Sorkin L. (2008). Effect of cytokines on neuronal excitability. *Neurosci. Lett.* 437 188–193. 10.1016/j.neulet.2008.03.052 18420346

[B54] SEQC/MAQC-III Consortium. (2014). A comprehensive assessment of RNA-seq accuracy, reproducibility and information content by the Sequencing Quality Control Consortium. *Nat. Biotechnol.* 32 903–914. 10.1038/nbt.2957 25150838 PMC4321899

[B55] Skosnik P. D. Cortes-Briones J. A. (2016). Targeting the ecology within: the role of the gut–brain axis and human microbiota in drug addiction. *Med. Hypotheses* 93 77–80. 10.1016/j.mehy.2016.05.021 27372861

[B56] Vallöf D. Vestlund J. Jerlhag E. (2019). Glucagon-like peptide-1 receptors within the nucleus of the solitary tract regulate alcohol-mediated behaviors in rodents. *Neuropharmacology* 149 124–132. 10.1016/j.neuropharm.2019.02.020 30772374

[B57] Waki H. Gouraud S. S. Maeda M. Paton J. F. R. (2010). Evidence of specific inflammatory condition in nucleus tractus solitarii of spontaneously hypertensive rats. *Exp. Physiol.* 95 595–600. 19923159 10.1113/expphysiol.2009.047324

[B58] Walker D. W. Hunter B. E. Riley J. (1975). A behavioral and electrophysiological analysis of ethanol dependence in the rat. *Adv. Exp. Med. Biol.* 59 353–372. 10.1007/978-1-4757-0632-1_25 1237223

[B59] Wang Y. Chiu W. Cheng C. Lee C. Chih W. H. (2021). A. Examination of neuroinflammatory cytokine interleukin-1 beta expression in the medial prefrontal cortex, amygdala, and hippocampus for the paradoxical effects of reward and aversion induced by morphine. *Neurosci. Lett.* 760:136076. 10.1016/j.neulet.2021.136076 34153368

[B60] Whitman B. A. Knapp D. J. Werner D. F. Crews F. T. Breese G. R. (2013). The Cytokine mRNA Increase Induced by Withdrawal from Chronic Ethanol in the Sterile Environment of Brain is Mediated by CRF and HMGB1 Release. *Alcohol. Clin. Exp. Res.* 37 2086–2097. 10.1111/acer.12189 23895427 PMC3815509

[B61] Williams C. L. Men D. Clayton E. C. Gold P. E. (1998). Norepinephrine release in the amygdala after systemic injection of epinephrine or escapable footshock: contribution of the nucleus of the solitary tract. *Behav. Neurosci.* 112 1414–1422. 10.1037/0735-7044.112.6.1414 9926823

[B62] Wilson J. S. Korsten M. A. Lieber C. S. (1986). The combined effects of protein deficiency and chronic ethanol administration on rat ethanol metabolism. *Hepatology* 6 823–829. 3530943 10.1002/hep.1840060504

[B63] Wolf Y. Yona S. Kim K. W. Jung S. (2013). Microglia, seen from the CX3CR1 angle. *Front. Cell. Neurosci.* 7:26. 10.3389/fncel.2013.00026 23507975 PMC3600435

[B64] Yang L. Wang M. Guo Y. Y. Sun T. Li Y. J. Yang Q. (2016). Systemic inflammation induces anxiety disorder through CXCL12/CXCR4 pathway. *Brain. Behav. Immun.* 56 352–362. 10.1016/j.bbi.2016.03.001 26952745

[B65] Zheng H. Cai L. Rinaman L. (2015). Distribution of glucagon-like peptide 1-immunopositive neurons in human caudal medulla. *Brain Struct. Funct.* 220 1213–1219. 10.1007/s00429-014-0714-z 24510283 PMC4127167

