# Supplementary figures and images for: Single Cell Scale Neuronal and Glial Gene Expression and Putative Cell Phenotypes and Networks in the Nucleus Tractus Solitarius in an Alcohol Withdrawal Time Series
Source: Front Syst Neurosci. 2021 Nov 19;15:739790. doi: 10.3389/fnsys.2021.739790 (PMC8641127; doi:10.3389/fnsys.2021.739790)

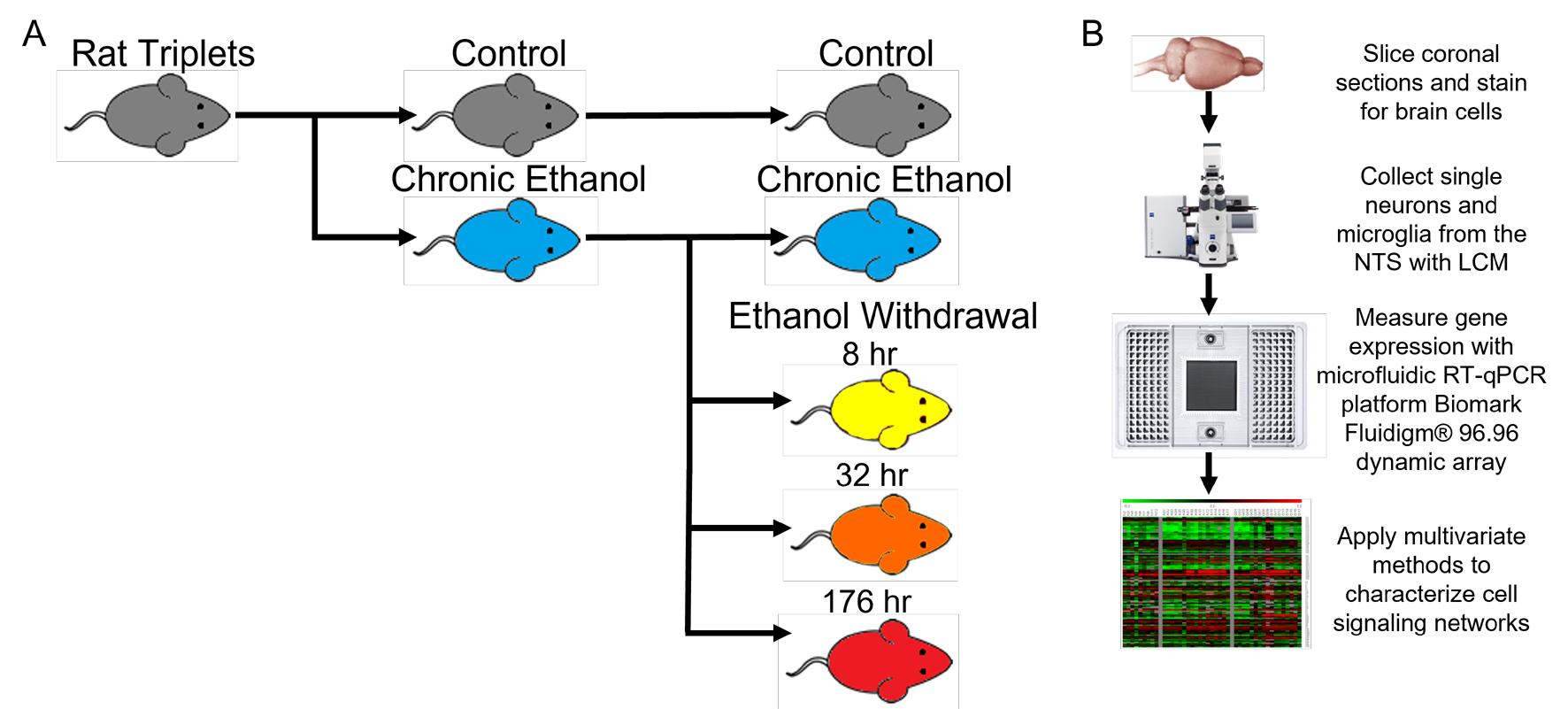

Supplement: Supplementary Figure 1 — Experimental design and single-cell selection. (A) Rat triplets were randomly assigned one of five treatments studied. (B) Single-cell selection and measure of transcription. [file Image_1.TIF]

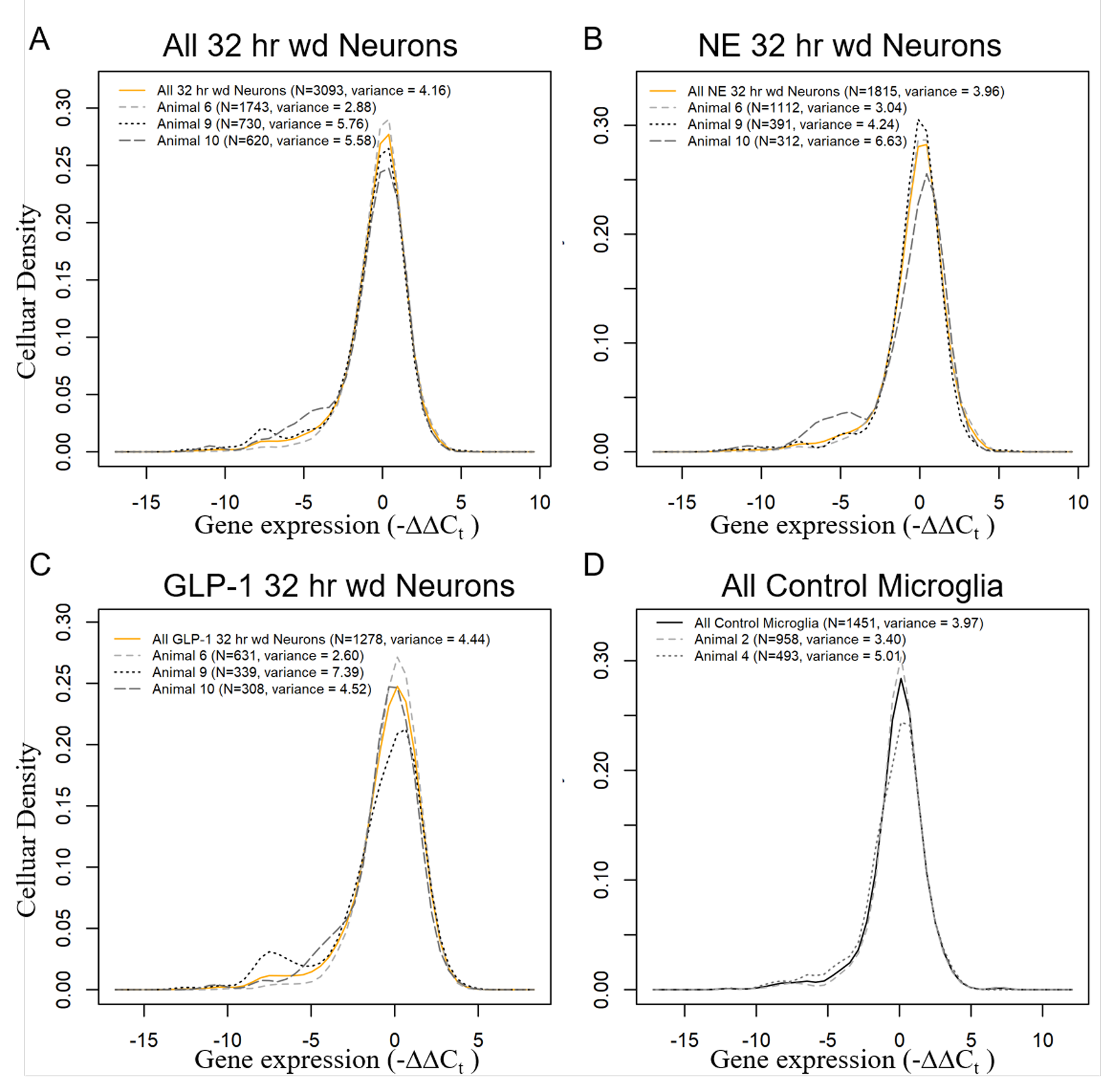

Supplement: Supplementary Figure 2 — Density plots of normalized data across all genes. (A) Three separate rats provided single-cell samples for 32 h wd neurons. Gene expression variance between animals was similar or less than gene expression variance within an animal. (All 32 h wd neurons N = 3093, variance = 4.16; Animal 6 N = 1743, variance = 2.88; Animal 9 N = 730, variance = 5.76; Animal 10 N = 620, variance = 5.58). (B) (All 32 h wd NE neurons N = 1815, variance = 3.96; Animal 6 N = 1112, variance = 3.04; Animal 9 N = 391, variance = 4.24, Animal 10 N = 312, variance = 6.63). (C) (All 32 h wd GLP-1 neurons N = 1278, variance = 4.44; Animal 6 N = 631, variance = 2.60, Animal 9 N = 339, variance = 7.39, Animal 10 N = 308, variance = 4.52). (D) Two separate rats provided single-cell samples for control microglia. Gene expression variance between animals was similar or less than gene expression variance within an animal (All Control Microglia N = 1451, variance = 3.97; Animal 2 N = 958, variance = 3.40; Animal = 4 N = 493, variance = 5.01). [file Image_2.TIF]

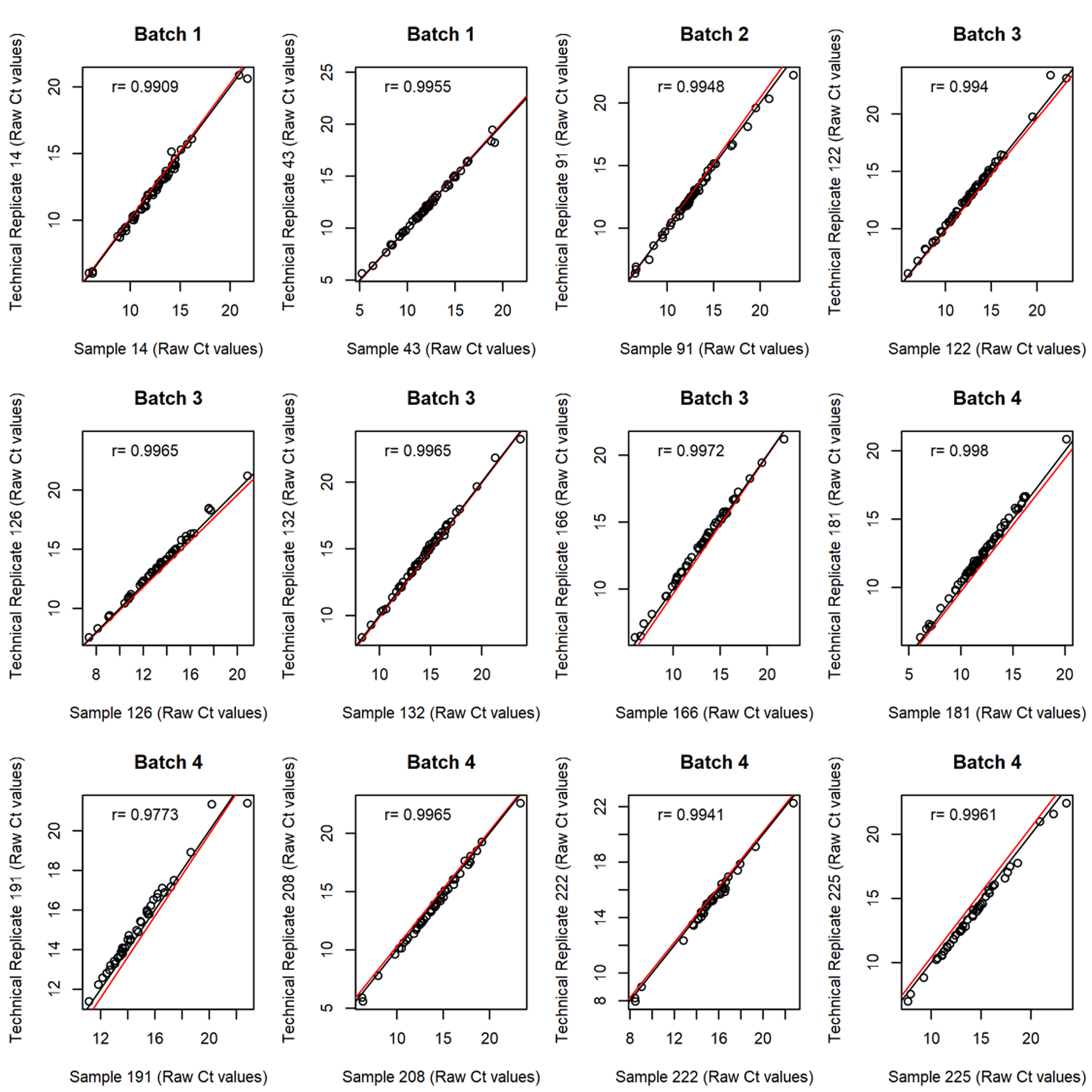

Supplement: Supplementary Figure 3 — Technical replicate plots of raw Ct values. [file Image_3.TIF]

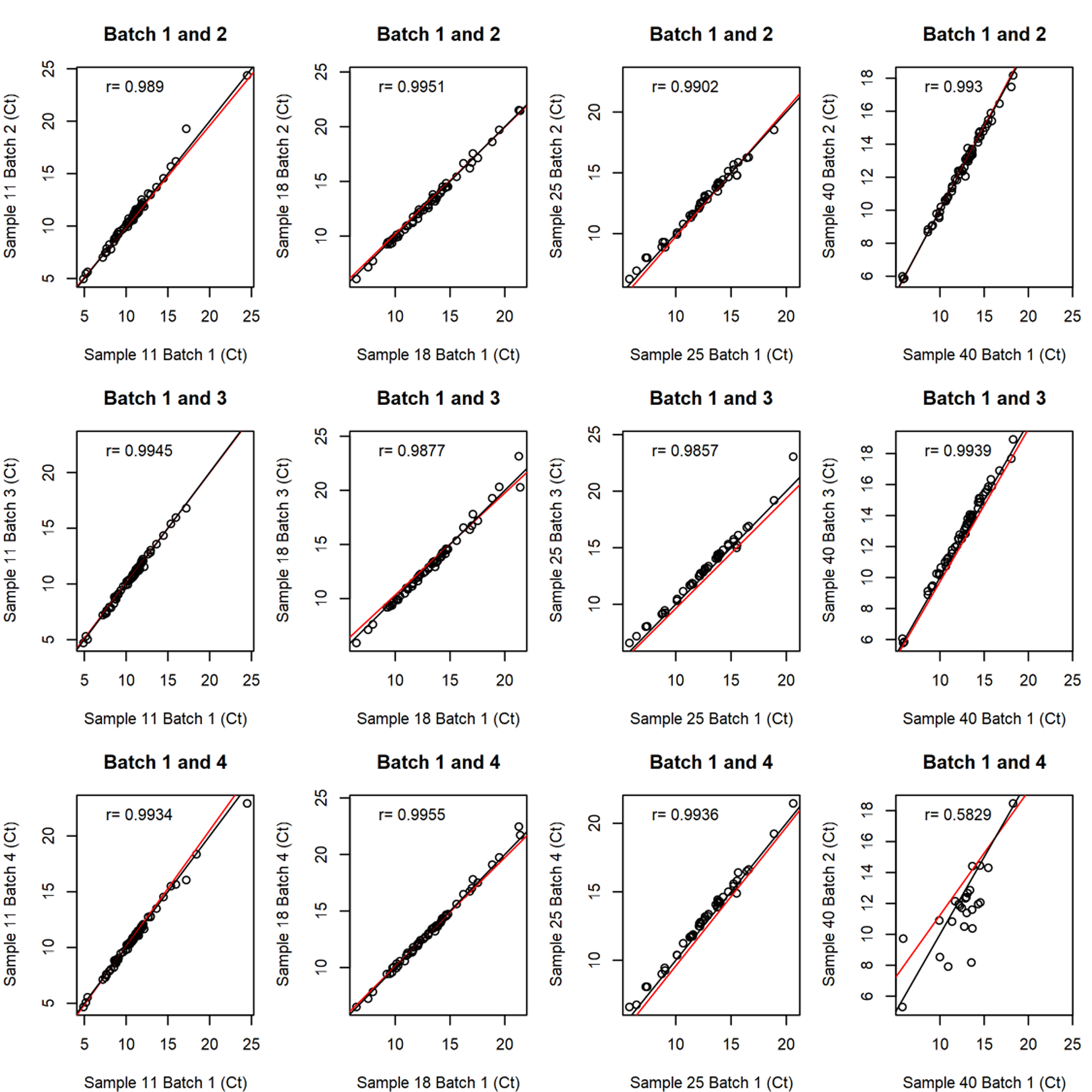

Supplement: Supplementary Figure 4 — Inter-batch replicate plots of raw Ct values. [file Image_4.TIF]

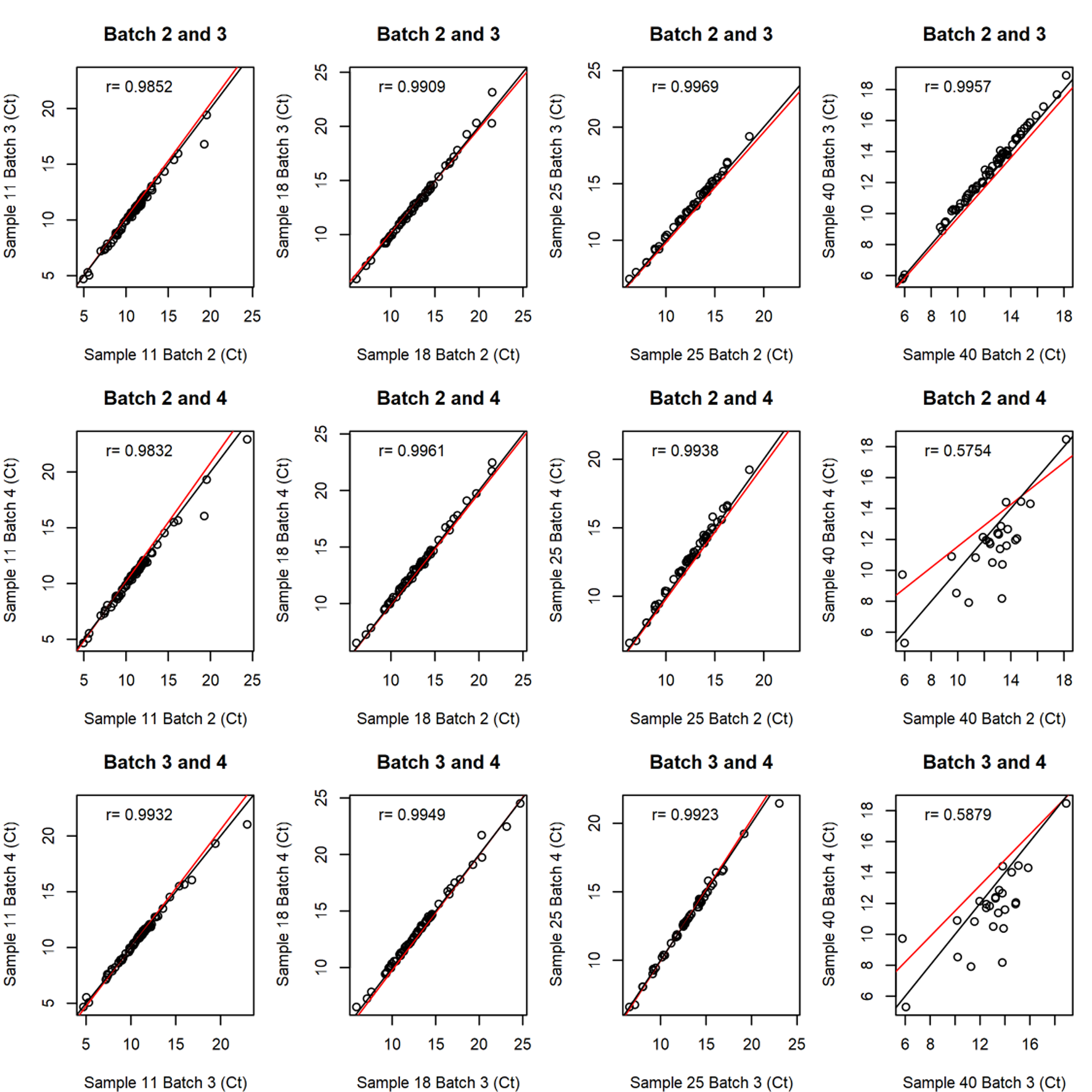

Supplement: Supplementary Figure 5 — Inter-batch replicate plots of raw Ct values. [file Image_5.TIF]

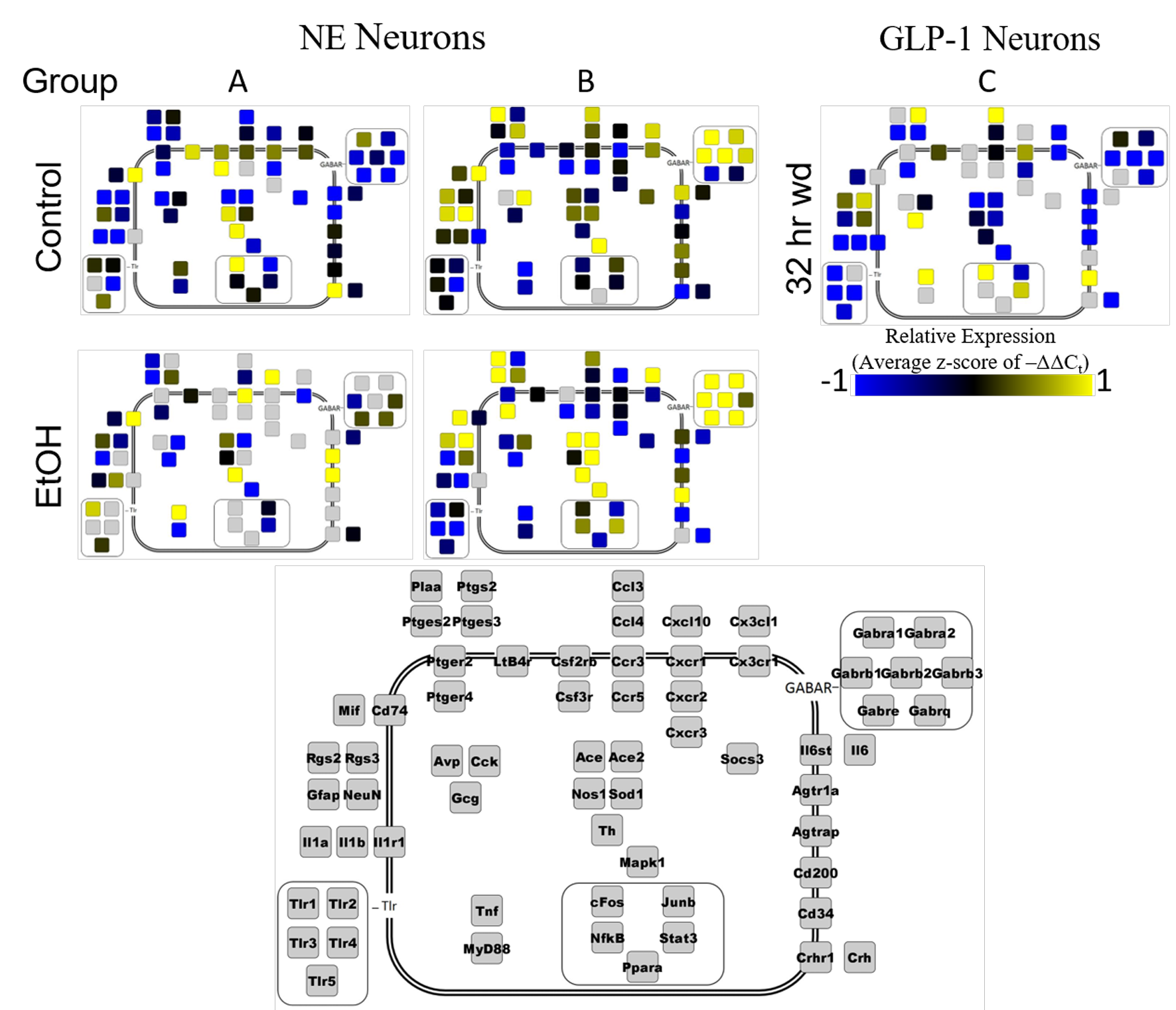

Supplement: Supplementary Figure 6 — Suphenotype gene expression in NE and GLP-1 neurons. Cellular cartoons display boxes representing relative gene expression (average z-score of −ΔΔCt values) of subphenotypes shown in prior heatmaps. Legend on right labels which boxes correspond to which gene and the color that represents expression (blue is low expression and yellow is high expression). The location of the box represents the localization or function of the protein product from that gene transcript. Legend is shown in gray boxes with labels. [file Image_6.TIF]

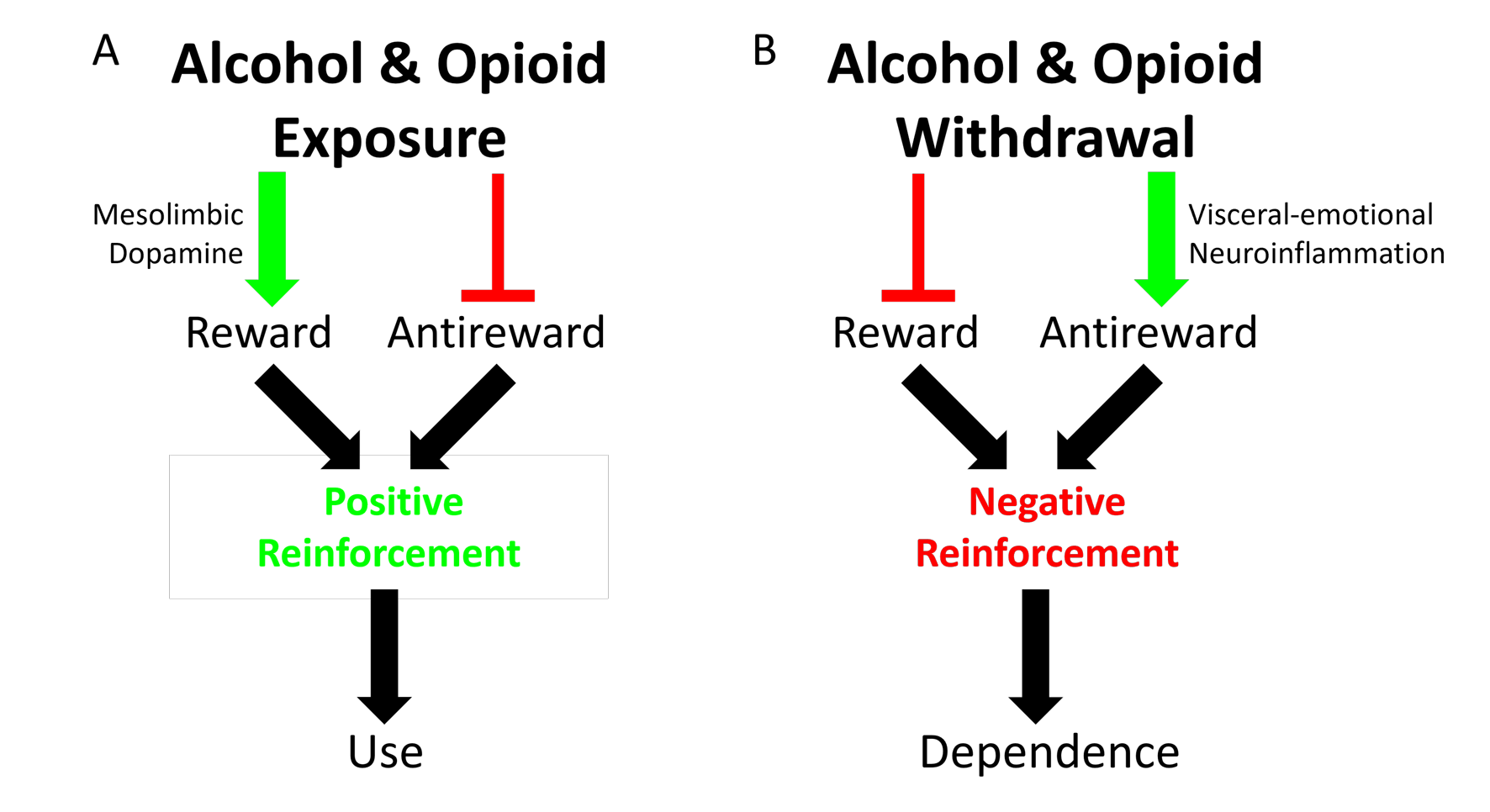

Supplement: Supplementary Figure 7 — Schematic of opponent-process model of addiction. Originally published in O’Sullivan and Schwaber (2021). (A) Alcohol and/or opioid exposure has two actions: Stimulate reward, via the mesolimbic dopamine pathway, and inhibit antireward. These actions motivate substance use via positive reinforcement. (B) Alcohol and/or opioid withdrawal has two action. Inhibit reward, by inhibiting the mesolimbic dopamine pathway (not shown), and stimulate antireward. This study proposes that visceral-emotional neuroinflammation is an endpoint in antireward stimulation, though this hypothesis warrants further testing. These actions, whatever the mechanism, motivate substance dependence via negative reinforcement. [file Image_7.TIF]
